# A Novel Benzoquinone Compound Isolated from Deep-Sea Hydrothermal Vent Triggers Apoptosis of Tumor Cells

**DOI:** 10.3390/md15070200

**Published:** 2017-06-26

**Authors:** Chenxi Xu, Xumei Sun, Min Jin, Xiaobo Zhang

**Affiliations:** College of Life Sciences and Laboratory for Marine Biology and Biotechnology of Qingdao National Laboratory for Marine Science and Technology, Zhejiang University, Hangzhou 310058, China; xuchenxi2013@126.com (C.X.); 21607053@zju.edu.cn (X.S.); jinmy136@zju.edu.cn (M.J.)

**Keywords:** deep-sea hydrothermal vent, thermophile, bacteriophage, benzoquinone, anti-tumor

## Abstract

Microorganisms are important sources for screening bioactive natural products. However, natural products from deep-sea microbes have not been extensively explored. In this study, the metabolites of bacteriophage GVE2 -infected (*Geobacillus* sp. E263 virus) thermophilic bacterium *Geobacillus* sp. E263, which was isolated from a deep-sea hydrothermal vent, were characterized. A novel quinoid compound, which had anti-tumor activity, was isolated from the phage-challenged thermophile. The chemical structure analysis showed that this novel quinoid compound was 2-amino-6-hydroxy-[1,4]-benzoquinone. The results indicated that 2-amino-6-hydroxy-[1,4]-benzoquinone and its two derivatives could trigger apoptosis of gastric cancer cells and breast cancer cells by inducing the accumulation of intracellular reactive oxygen species. Therefore, our study highlighted that the metabolites from the phage-challenged deep-sea microbes might be a kind of promising sources for anti-tumor drug discovery, because of the similarity of metabolic disorder between bacteriophage-infected microbes and tumor cells.

## 1. Introduction

Although remarkable advances in medicine have been achieved, cancer remains one of the most important causes of human mortality [1]. Among cancers, liver cancer is the second leading cause of cancer-related death in the world, while breast cancer is the leading cause of cancer death in women [2]. To treat cancers, many strategies have been developed. Tumor resection and chemotherapy are the most frequently used methods in the cancer treatment. In the early stage of cancers, tumor resection is effective. However, chemotherapy has severe side effects on patients. Thus, it is urgent to obtain compounds that target the cancer cells but have little effect on normal cells. Natural products have become one of important resources for discovery of anticancer drugs. As reported, more than 15,000 chemical substances including nearly 4000 bioactive marine natural products have been found from marine microorganism [3], indicating that marine microbes are important resources for anti-tumor compounds discovery. For example, Napyradiomycine derivatives, produced from a marine-derived *actinomyceteCNQ525*, are illustrated to induce apoptosis of colon adenocarcinoma cell line homo sapiens colorectal tumor cells 116 (HCT-116) [4]. *Gallinamide A*, isolated from a marine cyanobacterium, is shown to potently and selectively inhibit the human cysteine protease cathepsin L which is upregulated in multiple cancer cells [5]. Recently, Salinosporamide A, which is isolated from marine *Salinisporatropica*, was approved by FDA (Food and Drug Administration) of USA for treatment of multiple myeloma [6]. Although many bioactive compounds for anti-tumor treatment are obtained from marine bacteria, the anticancer metabolites from deep-sea microbes have not been extensively explored. As is well known, the deep sea possesses a lot of special ecosystems, such as deep-sea hydrothermal vents. In the vent ecosystem, chemolithoautotrophic microorganisms, which are capable of oxidizing hydrogen sulfide or other inorganic compounds to provide energy, are unique compared to other ecosystems [7,8]. The unique deep-sea vent ecosystem implies some unique metabolites may be produced by chemolithoautotrophic microbes.

Cancer is a disease involving multiple time- and space-dependent changes in the health status of cells and tissues that ultimately lead to malignant tumors. Emerging evidence indicates that cancer is a primarily metabolic disease [9]. One of the well-known phenomena about metabolic disorder found in cancers is the Warburg effect [10]. Proliferating tumor cells convert the majority of their glucose into lactate even in oxygen-rich conditions. As reported, during the bacteriophage–bacterium interactions, the bacterial metabolism is altered due to the virus infection [11]. In this context, there may be a relationship between tumor cell metabolic disorder and virus-induced host metabolic disorder. Our previous study showed that when the bacteria were challenged by bacteriophage, they could generate some special metabolites [12]. The bacteriophage-challenged bacterial metabolites may be an important resource of anticancer drugs. At present, however, this issue has not been extensively explored.

To evaluate the anti-tumor activity of metabolites from virus-induced bacteria, the thermophile *Geobacillus* sp. E263, which was isolated from a deep-sea hydrothermal vent [8], was challenged by its bacteriophage GVE2, followed by characterization of bioactive compounds from the virus-infected thermophile. The results revealed that a novel benzoquinone compound isolated from the GVE2-infected E263 presented its anti-tumor activity by triggering apoptosis of tumor cells.

## 2. Materials and Methods

### 2.1. Geobacillus *sp.* E263 Infection and Fermentation

The thermophilic *Geobacillus* sp. E263 strain from a deep-sea hydrothermal vent in East Pacific was infected by its bacteriophage GVE2. The E263 strain was challenged with the purified GVE2 virons at MOI (multiplicity of infection) of 5 when the OD_600_ (optical density at 600 nm) of the bacteria reached 0.3. After fermentation for 24 h at 60 °C, the bacteria were collected for metabolite extraction.

### 2.2. Extraction and Isolation of Bacterial Metabolites

The bacterial metabolites were extracted with methanol for 24 h at 4 °C. After three extractions, the supernatant was filter through a 0.45 μm filter. Then the filtrate was evaporated to dryness on a rotary evaporator under reduced pressure. The obtained crude extracts were resuspended into methanol. Subsequently, the solubilized metabolites were separated by semi-preparative HPLC (high performance liquid chromatography) using a C_18_ column and an H_2_O/CH_3_OH gradient (0~100% methanol for 60 min) by a UV (ultraviolet) detector at 254 nm.

### 2.3. Cell Proliferation Assay

The cell proliferation was determined with MTS (3-(4,5-dimethylthiazol-2-yl)-5-(3-carboxymethoxyphenyl)-2-(4-sulfophenyl)-2H-tetrazolium, inner salt) assay (Promega, Madison, WI, USA). Briefly, cells were seeded into a 96-well plate until they reached 60% confluence. The cells in each well were treated with a serially diluted compound. After incubation for 48 h, 20 μL MTS reagent was added to each well. Three hours later, the absorbance data at 450 nm of samples were recorded using a 96-well plate reader.

### 2.4. Identification of Isolated Compound and the Synthesis of Its Derivatives

To identify the isolated compounds, gas chromatography coupled mass spectrometry (GC-MS) was conducted as described previously [12]. Briefly, the compound was silylated with trimethylsilyl cyanide and then analyzed on a Q Exactive GC Mass Spectrometers (Thermo Scientific, Waltham, MA, USA). The obtained mass spectra were searched against the National Institute of Standards and Technology (NIST) database (New York, NY, USA). To reveal the structure of the isolated compound, NMR (Nuclear Magnetic Resonance) was carried out. ^1^H and ^13^C spectra were determined on a Bruker 500 MHz (Advance III DRX500, Billerica, MA, USA) spectrometer using DMSO-*d*_6_.

The derivatives were synthesized by Yuhao Chemical Company (Hangzhou, China).

### 2.5. Detection of Apoptosis

To detect apoptosis of cells treated with the compounds, 5000 cells per well were plated into a 96-well plate and incubated overnight at 37 °C. Then the compounds were added into cells. After incubation for 24 h, 100 μL Caspase-Glo reagent (Promega, Madison, WI, USA) was added into the cells, followed by incubation for 1 h at room temperature. Subsequently, the fluorescence of cells was determined using a GloMax 96 microplate reader (Promega).

### 2.6. Measurement of Intracellular ROS (Reactive Oxygen Species)

The intracellular ROS production was measured using CM-H_2_DCFDA (carboxy-2′,7′-di-chlorofluorescein) (Life Technology, Waltham, MA,USA) as a cell-permeant indicator of ROS. Breast cancer cells (MDA-MB-435) and gastric cancer cells (MGC-803) were seeded into a 6-well plate, followed by culture overnight. The cells were incubated with a compound at different concentrations or dimethylsulfoxide (DMSO) as a control for 24 h. Subsequently the cells were incubated with CM-H_2_DCFDA at room temperature for 30 min. The fluorescence of cells was detected by fluorospectrophotometer.

### 2.7. Statistical Analysis

The numerical data of three independent experiments were analyzed with one-way analysis of variation (ANOVA). Students’ *t*-test was employed to test the significant differences between different treatments.

## 3. Results

### 3.1. Novel Anti-Tumor Compound Isolated from Bacteriophage-Challenged Thermophileof Deep-Sea Hydrothermal Vent

**Figure 1:**
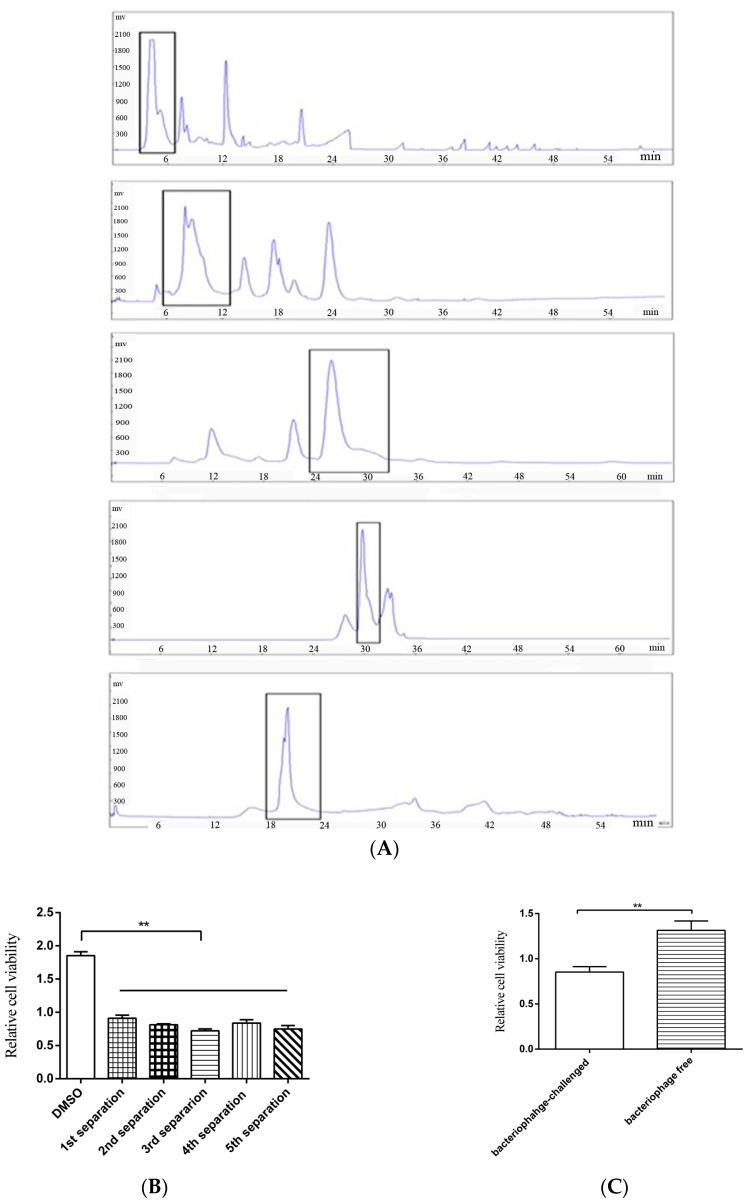

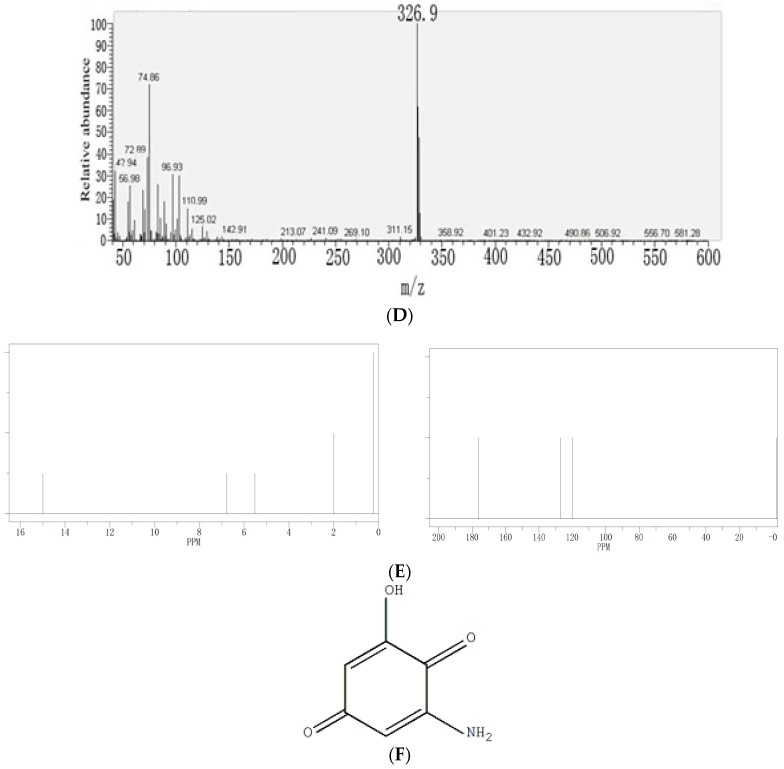
A novel anti-tumor compound isolated from bacteriophage-challenged thermophile from deep-sea hydrothermal vent. (**A**) The isolation of the anti-tumor compounds. The crude extracts from GVE2-infected *Geobacillus* sp. E263 were separated by semi-preparative HPLC using a H_2_O/CH_3_OH liner gradient. The compounds from each separation were subjected to cell proliferation assay. The peaks collected were indicated with boxes. (**B**) The anti-tumor activities of the isolated compounds. Breast cancer cells (MDA-MB-231) were treated with the isolated compounds. At 48 h after treatment, the cell viability was evaluated by cell proliferation assays using MTS. DMSO was included in the assays as a control. Data represented the mean ± standard deviation of triplicate. (**C**) The influence of crude metabolites from virus-challenged and virus-free bacteria on cancer cell proliferation. MDA-MB-231 cells were treated with extracted crude metabolites, followed by evaluation of cell viability. (**D**) GC-MS analysis of the isolated compound. After five successive separations, the isolated compound was subjected to GC-MS. The *m*/*z* of 326.97 indicated the compound with three trimethylsilyl. (**E**) ^1^H NMR (up) and ^13^C NMR (bottom) of the isolated compound. (**F**) The structure of the isolated compound. (** *p* < 0.01).

To obtain the anti-tumor compounds from bacteria, thermophile *Geobacillus* sp. E263 from a deep-sea hydrothermal vent was challenged with GVE2, followed by the extraction of virus-infected bacterial metabolites. The crude extracts of GVE2-challenged bacteria could suppress the growth of breast cancer cells (MDA-MB-231). To isolate the anti-tumor compounds, the crude bacterial metabolites were subjected to successive separations of metabolites and then the anti-tumor activity of metabolites were examined. After five successive separations of metabolites using semi-preparative HPLC, an anti-tumor compound was isolated (Figure 1A). This compound could inhibit the growth of breast cancer cells (MDA-MB-231) (Figure 1B). As a control, crude metabolites from bacteriophage-free bacteria were extracted and the influence of these metabolites on breast cancer cells (MDA-MB-231) was evaluated. The results indicated that the metabolites from virus-challenged bacteria significantly inhibited the cancer cell proliferation compared with those from virus-free bacteria (Figure 1C). To identify the isolated anti-tumor compound, the compound was subjected to GC-MS analysis (Figure 1D). The searching of the compound mass spectrum against the NIST database indicated that the isolated compound had no similarity to any known compounds, showing that the isolated compound was a novel compound. To determine the structure of this compound, ^1^H and ^13^C NMRs of the compound were conducted. The results revealed that the isolated compound was designated as 2-amino-6-hydroxy-[1,4]-benzoquinone, which contained a quinone ring (Figure 1E,F). The above findings indicated that a novel anti-tumor benzoquinone compound was isolated from deep-sea hydrothermal vent.

### 3.2. Effects of Derivatives of the Anti-Tumor Compound on the Tumor Cell Proliferation

**Figure 2:**
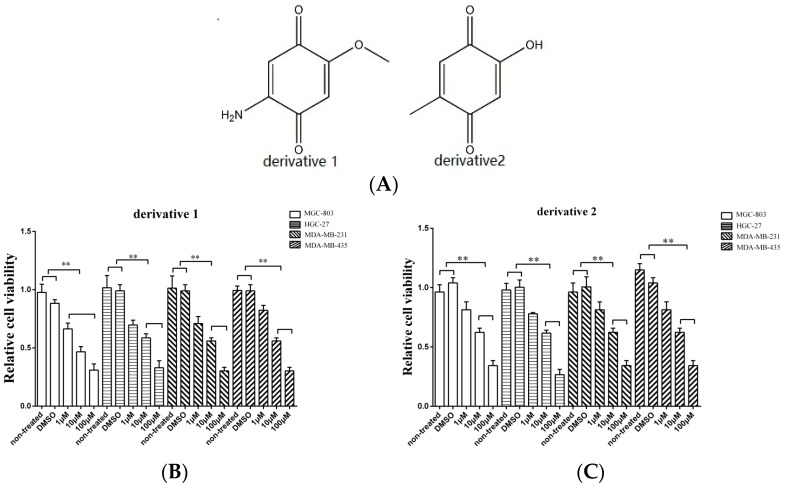
The effects of derivatives of the anti-tumor compound on the tumor cell proliferation. (**A**) The derivatives of the compound 2-amino-6-hydroxy-[1,4]-benzoquinone. (**B**,**C**) The effects of twoderivatives of 2-amino-6-hydroxy-[1,4]-benzoquinone on the cancer cells proliferation. The gastric cancer cells (HGC-27 and MGC-803), the breast cancer cells (MDA-MB-231) and the melanoma cells (MDA-MB-435) were treated with the derivatives at different concentration. At 48 h after treatment, the cell viability was evaluated. DMSO (1%) was included in the assays as a control. The viability of cells treated using 2-amino-6-hydroxy-[1,4] benzoquinone or its two derivatives was relative to that of the cells treated using DMSO. Data represented the mean ± standard deviation of triplicate assays (* *p* < 0.05; ** *p* < 0.01).

The isolated anti-tumor compound 2-amino-6-hydroxy-[1,4]-benzoquinone contains two important functional groups including a hydroxyl and an amino. In order to evaluate the roles of these functional groups in their function, two derivatives of 2-amino-6-hydroxy-[1,4]-benzoquinone were synthesized (Figure 2A). To assess the anti-tumor activity of the derivatives, gastric cancer cells (HGC-27 and MGC-803), breast cancer cells (MDA-MB-231), and melanoma cells (MDA-MB-435) were used. As shown in Figure 2B, when the cancer cells were treated with the two derivatives at 10 μM and 100 μM, the proliferation of cancer cells was significantly suppressed, indicating that the amino and hydroxyl groups did not contribute to the anti-tumor activity of 2-amino-6-hydroxy-[1,4]-benzoquinone.

Taken together, these findings revealed that 2-amino-6-hydroxy-[1,4]-benzoquinone and its two derivatives possessed anti-tumor activity against breast, gastric, and melanoma cancers.

### 3.3. Influence of Anti-Tumor Compound and Its Derivatives on Apoptosis of Tumor Cells

**Figure 3:**
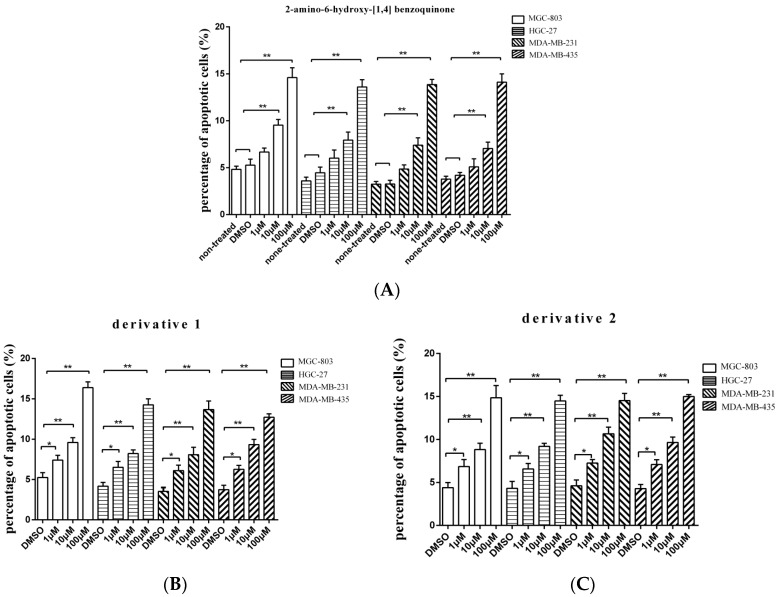
The influence of anti-tumor compound and its derivatives on apoptosis of tumor cells. (**A**) The effects of 2-amino-6-hydroxy-[1,4]-benzoquinone on apoptosis of cancer cells. The gastric cancer cells (HGC-27 and MGC-803), the breast cancer cells (MDA-MB-231) or the melanoma cells (MDA-MB-435) were treated with 2-amino-6-hydroxy-[1,4]-benzoquinone at different concentrations. Twenty four hours later, the caspase 3/7 activity of cancer cells was examined. (**B**) The influence of derivative 1 on apoptosis of cancer cells. The cancer cells were incubated with derivative 1 for 24 h and then the caspase 3/7 activity was evaluated. (**C**) The role of derivative 2 in cancer cell apoptosis. After incubation with derivative 2 for 24 h, the caspase 3/7 activity of cancer cells was assessed. In all panels, the data represented means ± standard deviations of triplicate assays (* *p* < 0.05; ** *p* < 0.01).

To evaluate whether the isolated compound 2-amino-6-hydroxy-[1,4]-benzoquinone and its two derivatives induced the gastric, breast, and melanoma cancer cell apoptosis, the caspase 3/7 activities of cancer cells treated with the compounds at different concentrations were examined. The results showed that the percentage of apoptotic cancer cells treated with 2-amino-6-hydroxy-[1,4]-benzoquinone at 10 or 100 μM was significantly increased compared with the controls (Figure 3A), while the two derivatives induced apoptosis of cancer cells at lower concentration (1 μM) (Figure 3B,C). Among the three compounds, derivative 1 presented the highest activity to induce apoptosis of cancer cells (Figure 3B). These findings revealed that 2-amino-6-hydroxy-[1,4]-benzoquinone and its two derivatives could trigger apoptosis of cancer cells.

### 3.4. Mechanism of Anti-Tumor Compound-Induced Apoptosis of Tumor Cells

**Figure 4:**
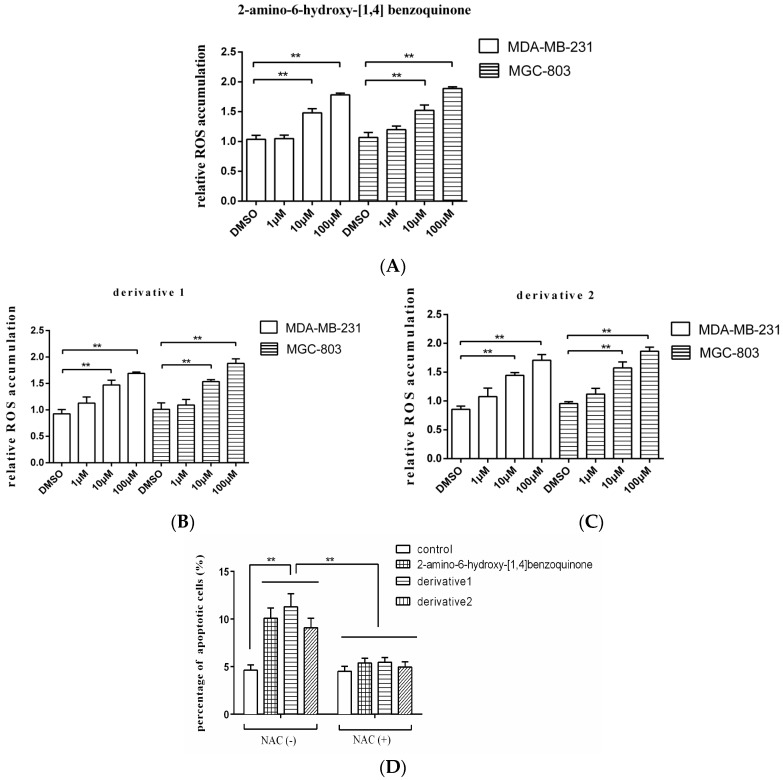
The mechanism of anti-tumor compound-induced apoptosis of tumor cells. The breast cancer cells (MDA-MB-231) or gastric cancer cells (MGC-803) were treated with the isolated anti-tumor compound 2-amino-6-hydroxy-[1,4]-benzoquinone (**A**) and its two derivates (**B**,**C**) at different concentrations. Twenty-four hours later, the ROS accumulation in the cells was examined with fluorospectrophotometer. The ROS accumulation of the cells treated with 2-amino-6-hydroxy-[1,4]-benzoquinone or its two derivatives was relative to that of the cells treated with DMSO. (**D**) The role of ROS in apoptosis of gastric cells. Gastric cancer cells (MGC-803) were treated 2-amino-6-hydroxy-[1,4]-benzoquinone or its two derivatives in the presence or absence of NAC (*N*-acetyl-l-cysteine) for 24 h. Then the apoptotic cells were examined. The control was the cells treated with DMSO. In all panels, the data were shown as means ± standard deviations (*n* = 3). The statistical difference significance between treatments were indicated with asterisks (** *p* < 0.01).

As reported, the quinone-like compound can induce oxidative stress and ROS (reactive oxygen species) accumulation in cells, then leading to apoptosis [13]. In order to explore the mechanism of apoptosis induced by anti-tumor compound, therefore, the ROS accumulation in the tumor cells treated with 2-amino-6-hydroxy-[1,4]-benzoquinone and its two derivatives was examined. The results showed that the ROS accumulations in breast cancer cells (MDA-MB-231) and gastric cancer cells (MGC-803) were significantly increased by the anti-tumor compound 2-amino-6-hydroxy-[1,4]-benzoquinone at 10 and 100 μM compared with the DMSO control (Figure 4A), which further triggered apoptosis of cancer cells. The two derivatives of 2-amino-6-hydroxy-[1,4]-benzoquinone yielded the similar results (Figure 4B,C). To explore the role of ROS in apoptosis, gastric cancer cells (MGC-803) were treated with 2-amino-6-hydroxy-[1,4]-benzoquinone or its two derivatives and NAC (*N*-acetyl-l-cysteine), an antioxidant which functions as a ROS scavenger [14], followed by the detection of apoptosis. It was revealed that 2-amino-6-hydroxy-[1,4]-benzoquinone and its two derivatives could not induce apoptosis of MGC-803 cells in the presence of NAC compared with the control (Figure 4D), indicating that ROS played a positive role in the induction of apoptosis.

Taken together, these findings presented that the anti-tumor compound 2-amino-6-hydroxy-[1,4]-benzoquinone and its two derivatives triggered apoptosis of cancer cells by inducing the ROS accumulation in cancer cells.

## 4. Discussion

Cancer is a leading cause that threatens human health. Except for tumor resection on early stage of tumor formation, the treatment of tumor with specific compounds that target the tumor cells is an important strategy for cancer therapy [15]. Both natural products and synthesized compounds are vital sources for the discovery of invaluable anti-tumor compounds [16]. Especially from the natural products, many structurally special and functionally effective anti-tumor compounds have been found. At present, natural products from microbes represent a very important source for discovering anti-tumor compounds [17]. Deep-sea hydrothermal vents, very special ecosystems in oceans, are enriched in microorganisms [8]. In the deep-sea vent ecosystems, thermophilic chemosynthetic prokaryotes exploit the vent chemicals to obtain energy for their growth, and the vent animals in the environment, such as tubeworms, huge clams, crabs, and several species of fish, are supported by on-site production by chemosynthetic thermophiles. As important agents for thermophile mortality, bacteriophages infecting thermophiles are believed to be the major players in the deep-sea vent ecosystems. Due to the similarity of metabolic disorder between bacteriophage-infected microbes and tumor cells, the metabolites from the bacteriophage-challenged microorganisms that reside in deep sea hydrothermal vents may be a resource for screening anti-tumor compounds. In this study, the results indicated that 2-amino-6-hydroxy-[1,4]-benzoquinone obtained from the GVE2-infected thermophilic bacterium *Geobacillus* sp. E263 possessed anti-tumor activity. Thus, our study revealed that the metabolites of the bacteriophage-challenged deep-sea vent thermophiles were important resources for discovering novel anti-tumor drugs.

Our study showed that 2-amino-6-hydroxy-[1,4]-benzoquinone exhibited anti-tumor activity against breast cancer, gastric cancer, and melanoma by triggering apoptosis of the cancer cells. It is well-known that quinone compounds such as daunorubicin and doxorubicin can form reactive oxygen species (ROS) to induce cells to apoptosis [18]. The high concentration of ROS in cells promotes the cellular senescence and apoptosis [19]. In this investigation, the anti-tumor compound 2-amino-6-hydroxy-[1,4]-benzoquinone, belonging to the quinone class of organic compounds family, could induce the accumulation of ROS in breast and gastric cancer cells. As reported, the intracellular ROS can trigger oxidative stress and increase the activity of the antioxidant defense system to cause the mitochondrial damage [20]. The ROS accumulation in cells can permeabilize the mitochondrial membrane and induce the leakage of proteins to cells to activate the caspase 9 cascade, leading to apoptosis of breast and gastric cancer cells in a mitochondrial-dependent pathway [13]. In this study, therefore, the ROS accumulation in 2-amino-6-hydroxy-[1,4]-benzoquinone-treated cancer cells might trigger the mitochondrial apoptotic pathway of cancer cells. Besides, the ROS production of breast and gastric cancer cells treated with 2-amino-6-hydroxy-[1,4]-benzoquinone might be associated with the redox cycling of quinone-containing substances. During the redox recycling, quinoid substances in the reduced states can yield extra electrons to oxygen with the formation of superoxide anions, beginning a cascade that generates H_2_O_2_ and hydroxyl radicals [21]. In this context, 2-amino-6-hydroxy-[1,4]-benzoquinone might alter the redox state of cancer cells and breakthe balance of redox states to cause the cancer cells to death. To further reveal the mechanism of 2-amino-6-hydroxy-[1,4]-benzoquinone-induced apoptosis of cancer cells, the proteins interacted with 2-amino-6-hydroxy-[1,4]-benzoquinone merited to be explored in the future study. If the target proteins of 2-amino-6-hydroxy-[1,4]-benzoquinone were upregulated in cancer cells compared with normal cells, 2-amino-6-hydroxy-[1,4]-benzoquinone could only trigger apoptosis of cancer cells.
